# Molecular and Neuroendocrine Approaches to Understanding Trade-offs: Food, Sex, Aggression, Stress, and Longevity—An Introduction to the Symposium

**DOI:** 10.1093/icb/icx113

**Published:** 2017-09-11

**Authors:** Jill E Schneider, Pierre Deviche

**Affiliations:** 1 Department of Biological Sciences, Lehigh University, 111 Research Drive, Bethlehem, PA 18015, USA; 2 School of Life Sciences, Arizona State University, Tempe, AZ 85287–4501, USA

## Abstract

Life history strategies are composed of multiple fitness components, each of which incurs costs and benefits. Consequently, organisms cannot maximize all fitness components simultaneously. This situation results in a dynamic array of trade-offs in which some fitness traits prevail at the expense of others, often depending on context. The identification of specific constraints and trade-offs has helped elucidate physiological mechanisms that underlie variation in behavioral and physiological life history strategies. There is general recognition that trade-offs are made at the individual and population level, but much remains to be learned concerning the molecular neuroendocrine mechanisms that underlie trade-offs. For example, we still do not know whether the mechanisms that underlie trade-offs at the individual level relate to trade-offs at the population level. To advance our understanding of trade-offs, we organized a group of speakers who study neuroendocrine mechanisms at the interface of traits that are not maximized simultaneously. Speakers were invited to represent research from a wide range of taxa including invertebrates (e.g., worms and insects), fish, nonavian reptiles, birds, and mammals. Three general themes emerged. First, the study of trade-offs requires that we investigate traditional endocrine mechanisms that include hormones, neuropeptides, and their receptors, and in addition, other chemical messengers not traditionally included in endocrinology. The latter group includes growth factors, metabolic intermediates, and molecules of the immune system. Second, the nomenclature and theory of neuroscience that has dominated the study of behavior is being re-evaluated in the face of evidence for the peripheral actions of so-called neuropeptides and neurotransmitters and the behavioral repercussions of these actions. Finally, environmental and ecological contexts continue to be critical in unmasking molecular mechanisms that are hidden when study animals are housed in enclosed spaces, with unlimited food, without competitors or conspecifics, and in constant ambient conditions.

## Introduction

Life is full of trade-offs in which organisms must forfeit something of value to gain something new. Resources such as time and energy are often finite, and thus, engagement in some activities often excludes engagement in others. Careful attention to constraints and trade-offs can help us elucidate the mechanisms that underlie variation in life history strategies. For example, energetic and time constraints might underlie some of the seasonal, estrous/menstrual, and circadian cycles observed in phenotypes related to both reproduction and energy metabolism. With regard to annual cycles, many warm-blooded species that inhabit temperate zones must reconcile the benefits of immediate reproduction with the costs of high offspring and parent mortality at low ambient temperatures, particularly when cold temperatures are accompanied by low food availability. This group of symposium papers includes new and reviewed information about the trade-offs in seasonally breeding, temperate zone bats (Willis 2017), voles (Ferkin 2017), hamsters (Schneider et al. 2017), birds (Deviche et al. 2017), and snakes (Lucas et al. 2017). In many such species, reproductive processes are suppressed to conserve energy for thermogenesis, and the hypothalamic–pituitary–gonadal (HPG) system is inhibited in direct response to the resulting energy deficits or to indirect cues that predict the onset of winter (reviewed by Bairlein and Gwinner 1994; Bronson 1989). Similar environmental cues can promote the onset of reproductive processes in the spring. For example, in this symposium, we learn that in some bat species, one important cue for initiation of the breeding season is an abrupt drop in barometric pressure within the hibernaculum, which heralds a warm front outside the cave (Willis 2017). Additional trade-offs occur in species that show annual cycles in hibernation or migration. Both of these strategies provide the benefit of avoiding costs associated with cold ambient temperatures and/or low food availability, but have associated costs, such as compromised immune function and/or loss of restorative sleep (e.g., Willis 2017 and reviewed by Alerstam and Lindstrom 1990). Specific interactions between environmental cues and neuroendocrine systems create important species and sex differences in seasonal life history strategies (e.g., reviewed by Bentley et al. 2017; Davies and Deviche 2014; Ferkin 2017; Lucas et al. 2017, in this symposium). In addition to annual cycles, estrous/menstrual cycles are associated with trade-offs. The energetic costs of reproduction require that periods of sexual activity be preceded by periods of foraging, eating, body fat storage, and/or food hoarding. This is particularly important in female mammals living in habitats in which energy supply and demand fluctuate. The fluctuations in ingestive and reproductive activities synchronize mating with ovulation, and might link high levels of sexual motivation with a suppressed appetite for food. Presentations in this symposium illustrate how availability of food and other stressors inhibit many aspects of reproduction, including gonadotropin and gonadal steroid synthesis, gamete production (Bentley et al. 2017; Deviche et al. 2017) attractivity, proceptivity, and receptivity (Ferkin 2017), and simultaneously increases ingestive behaviors, including foraging and food hoarding (Schneider et al. 2017). The link between ingestive and sex behavior might provide important clues to underlying mechanisms, clues that would not be likely to arise from the more common assumption that “satiety hormones” should maintain body weight in individuals isolated from opposite-sex conspecifics, in an enclosed space, with few behavioral options, and with unlimited food (reviewed by Schneider et al. 2013; Schneider et al. 2017). In addition to annual and ovulatory cycles, circadian cycles are associated with trade-offs. In at least some cases, time and/or energy constraints and their resulting trade-offs underlie circadian fluctuations in behavior that might synchronize activities such as dispersal, foraging, mating, and resting/energy conservation (Zera 2016a). In summary, studies of annual, estrous/menstrual, and circadian cycles have formed a strong foundation for understanding mechanistic trade-offs made by individuals.

Trade-offs are important at the level of the individual, but in addition, some trade-offs influence the process of evolution. This occurs when there is a cost in fitness associated with increases in an otherwise adaptive phenotype (Williams 1966; Stearns 1989; Zera and Harshman 2001). A microevolutionary trade-off can be defined as a negative relationship between two traits that prevents each trait from evolving independently (Stearns 1989). For example, in some species, high levels of fecundity are incompatible with longevity (Jenkins et al. 2004; Mukhopadhyay and Tissenbaum 2007). In other species, high levels of fecundity are incompatible with phenotypes that are necessary for dispersal (Zera and Denno 1997). In some of these cases, the evolution of two phenotypes is constrained because both phenotypes depend upon a limited resource, for example, when there is insufficient availability of metabolic fuels to support both the immune and reproductive system (Zera and Harshman 2001). Furthermore, constraints can be genetic, developmental, morphological, or physiological (Stearns 1989; Harshman and Zera 2007; Heideman and Pittman 2009; Ross et al. 2009; Heideman et al. 2010). There is a growing body of excellent research on the underlying physiology of trade-offs (e.g., Cox et al. 2009, 2010; Denver et al. 2009; Clark et al. 2015; Kirschman et al. 2017; Zera 2016a; Zera et al. 2016) as well as careful work that uncovers species that employ flexible strategies instead of making the expected trade-offs (Merrill et al. 2015). Yet, there remains a lack of evidence rigorously supporting even some of the most fundamental aspects of trade-off physiology—such as the very existence of purported trade-offs and the mechanisms involved.

One promising line of investigation involves components of the neuroendocrine system. This line of work demonstrates that the opposing pleiotropic effects of hormones impose constraints on the evolution of individual phenotypes that are affected by these hormones (Ketterson and Nolan 1992; Finch and Rose 1995; Hau and Wingfield 2011). Some prominent examples illustrate purported trade-offs that appear to be hormonally-mediated and, in addition, pique some critical questions. For example, in the brown anole lizard, *Anolis sagrei*, maximum longevity is incompatible with high levels of fecundity. In support of this idea, longevity is significantly increased by removal of ovarian hormones (by ovariectomy) concomitant with higher levels of growth, body fat content, hematocrit, immune response, and parasite tolerance (Cox et al. 2010). In many bird species, high levels of androgens promote territorial aggression and mating, but chronically elevated plasma concentrations of androgens incur severe costs in terms of immune function, survival rate, and parental care (reviewed by Wingfield et al. 1990; Hau and Wingfield 2011). Wing polymorphic *Gryllus* crickets include morphs that trade-off fecundity for dispersal capabilities related to large wing size and highly developed flight muscles. A long-winged morph has flight capability, high levels of triglycerides (presumably for storage of flight fuel), and high levels of acoustic sensitivity coupled with circadian rhythms in levels of juvenile hormone that underlie daily dispersals. Long-winged females tend to delay ovarian growth and egg production, whereas short-wing females have faster rates of ovarian development and higher fecundity related to chronically high concentrations of insulin-like peptides and ecdysteroids (reviewed by Zera 2016a, 2016b). These landmark studies demonstrate the potential role of hormones in trade-offs in both wild and laboratory animals, and they delineate some interesting and fundamental questions that remain unresolved in most systems: What are the cause-and-effect relations among hormones, metabolic pathways, metabolic fuel partitioning, behavior, and measures of reproductive success? For example, do gonadal steroids alter fuel oxidation and reproductive behavior and thereby “waste” energy that could be funneled toward longevity (e.g., fighting parasitic infections) and vice versa? More specifically, do the hormones involved in uptake and/or metabolism of carbohydrates redirect fuels toward tissues involved in reproductive output? New evidence suggests that symbiotic microorganisms in the intestines can increase or decrease calorie and nutrient uptake. Do hormones of the HPG system alter the gut microbiome to allow the individual to absorb more or less calories and nutrients from digested food during different reproductive stages? In addition, do gonadal steroids promote risky behaviors and social interactions that increase the incidence of infection with bacteria, viruses, and parasites? We envision two general possibilities: (1) energy allocated to one trait compromises survival by competing for a common pool of metabolic fuels or other resources necessary for growth, survival, and/or for other phenotypes such as mounting an immune response, or (2) pleiotropic effects of hormones or rhythms in hormones might, in and of themselves, constrain the evolution of the traits affected by the hormones. Are the two possibilities mutually exclusive? Could they both be simultaneously true? In reality, there might be overlap in and interaction among the different constraints. One possible example would occur when (1) two behaviors are influenced in opposite directions by the level of available metabolic fuels, and (2) the actions of hormones and metabolic intermediates increase or detract from the availability of internal resources (the overall pool of available metabolic fuels) thereby creating the stimulus for one or the other conflicting behavior. Furthermore, different types of trade-offs are made at the level of the individuals, populations, and species (e.g., Brozek et al. 2017; Crespi and Travis 2017); are the mechanisms of these types of trade-off the same, different, overlapping, and/or interacting (Stearns 1989)? Physiology, animal behavior, and life history strategy have been loosely associated with the idea of trade-offs, and yet, we have much to learn about the molecular and neuroendocrine mechanisms that create trade-offs.

Our symposium speakers are interested in the impact of trade-offs on individuals, families, and populations. The symposium presentations ranged from highly molecular (e.g., the presentation by Scott Emmons on the nematode worm, *Caenorhabditis elegans*, a species for which all of the genes and neural synapses are known) to highly behavioral (e.g., the presentation by Michael Ferkin on meadow voles, *Microtus pennsylvanicus*, a species in which new behavioral and physiological trade-offs have been recently characterized). Emmons explored the trade-off made by males of the species, *C. elegans*, between survival at a plentiful food source and reproductive success that becomes possible only upon leaving a food source. His laboratory group was able to identify genes that prioritize sexual and ingestive motivation, and to localize changes in gene expression in the nervous system. Erica Crespi explored the trade-off between offspring size and offspring number in killifish born into environments with different population densities and predation risk, and studied the mediation of this trade-off by progesterone (E. J. Crespi, unpublished data). Deborah Lutterschmidt presented her work on the neuroendocrine mechanisms that orchestrate seasonal patterns in reproductive behavior and ingestive behavior in red-sided garter snakes, *Thamnophis sirtalis parietalis*. This species hibernates in winter, emerges in spring, immediately engages in a two-week period of fasting and frenzied mating behavior, and then abruptly reverses priorities as it migrates to the feeding grounds. These behavioral transitions are associated with changes in a number of chemical messengers including arginine vasotocin and neuropeptide Y (NPY) (Lucas et al. 2017). Pierre Deviche reviewed the diversity of neuroendocrine mechanisms involved in the allocation of resources in birds with emphasis on the HPG and hypothalamic-pituitary-adrenal (HPA) systems (Deviche et al. 2017). George Bentley used examples from avian and mammalian species to illustrate the fact that one chemical messenger can have different effects centrally and peripherally creating species, sex, and developmental variation in the mechanisms involved in trade-offs (Bentley et al. 2017). Greg Demas presented the trade-off between reproduction and survival/longevity in the context of the energetic costs of the immune response and the energetic savings of sickness behaviors in Siberian hamsters (*Phodopus sungorus*). Craig Willis presented the unique perspective on energy allocation that comes from studying species that hibernate, with emphasis on little brown bats (*Myotis lucifugus*). Michael Ferkin (studying voles, *M. pennsylvanicus*) and Jill Schneider (studying Syrian hamsters, *Mesocricetus auratus*) discussed the importance of understanding the neuroendocrine control of both appetitive and consummatory behaviors for understanding trade-offs between reproduction and survival when environmental energy is limiting (Ferkin 2017; Schneider et al. 2017). At least three contributors discussed inter-generational trade-offs that include gestational programming, offspring development, and adult energy balancing traits (Brozek et al. 2017; Crespi and Travis 2017; Ferkin 2017). Using killifish (*Heterandria formosa*) as a model system, Crespi and Travis explored the expected trade-off between offspring size and number in response to population density/predation threat. Jeremy Brozek discussed maternal programming of offspring energy balancing characteristics, which might be adaptive when the offspring are born into the same environment in which they were programmed, but might force a trade-off if offspring are born into a different, mismatched environment (Brozek et al. 2017). Ferkin discovered that vole dams that are food restricted during lactation spend less time nursing and licking their offspring compared with control dams, and low rates of maternal licking during early and middle lactation had permanent effects on offspring that lasted into adulthood. Adult offspring of lactationally food-restricted dams showed reduced levels of attractivity, proceptivity, and receptivity, which are likely to influence their reproductive success (Ferkin 2017). All of the contributors are working on different physiological systems, including the stress response (Bentley et al. 2017), hibernation (Willis 2017), immunity (Crespi and Travis 2017), ingestive behavior (Brozek et al. 2017; Crespi and Travis 2017; Emmons 2017; Ferkin 2017; Lucas et al. 2017; Schneider et al. 2017), and reproduction (Brozek et al. 2017; Crespi and Travis 2017; Deviche et al. 2017; Emmons 2017; Ferkin 2017; Lucas et al. 2017; Schneider et al. 2017). Not all contributors to this symposium are testing specific hypotheses about microevolutionary trade-offs, and many of the contributors are primarily concerned with neuroendocrine mechanisms. At the same time, they are sincerely interested in both proximate and ultimate influences on behavior, and their work is likely to have something important to contribute to our understanding of trade-offs. In viewing this diverse array of model systems together as a whole, some important themes emerge.

## Moving beyond endocrine secretions

Certainly, hormones and their receptors play a role in orchestrating trade-offs (Adkins-Regan 2005). For example, trade-offs between territoriality and parental investment appear to be linked to polymorphisms in the estrogen receptor (Huynh et al. 2011; Horton et al. 2012, 2013). It is important to remember, however, that the definition of a hormone is a human invention, and there is little evidence that evolutionary forces, such as natural selection, abide by this definition. It is clear that we must move beyond traditional endocrine secretions to consider a more inclusive array of chemical messengers. In addition to peripheral hormones, neuropeptide hormones, neurotransmitters, and their receptors, the list of chemical factors that influence trade-offs includes immune secretions, such as growth factors, cytokines, chemokines, neurotrophins, complement factors, prostaglandins, metabolic intermediates, and enzymes, including aromatase. Some enzymes influence trade-offs by their effects on hormone synthesis, whereas others moonlight as signal transducers. Many different types of chemical messengers can be altered by environmental stimuli and can act as signaling molecules that send information from cell to cell. Like hormones, they as well can orchestrate trade-offs.

In its simplest form, a trade-off implies that an increase in one trait leads to a decrease in another, and many chemical messengers can stimulate one physiological process while inhibiting another. For example, each member of a growing list of chemical messengers increases food intake while simultaneously inhibiting reproductive processes. The chemical messengers that increase ingestive behaviors and inhibit reproductive processes include ghrelin and (at high circulating levels) insulin. In addition, the list includes a rapidly growing list of neuropeptides and monoamines. Our symposium highlighted gonadotropin-inhibition hormone (GnIH) and its mammalian ortholog, RFAmide-related peptide-3 (RFRP-3), in talks by Bentley and Schneider. Lutterschmidt emphasized arginine vasotocin and NPY. Other investigators have emphasized ghrelin, agouti-related protein, melanin-concentrating hormone, serotonin, and β-endorphin. Conversely, when food availability is abundant, ingestive behaviors are decreased by chemical messengers of the endocrine system such as leptin, the adipocyte hormone studied by at least four speakers in our symposium (Emmons, Lutterschmidt, Schneider, and Willis). Other anorexigenic molecules include glucagon-like peptide, cholecystokinin, estradiol, kisspeptin, dopamine, and α-melanocyte stimulating hormone. Many of these chemical messengers stimulate HPG function, courtship and copulatory behavior, maternal behavior, growth, maturation, and many aspects of the immune response, and many are involved in seasonal cycles of feeding and breeding (Hobbs et al. 2012; Schneider et al. 2013; Kriegsfeld et al. 2015; Ashley and Demas 2017; Deviche et al. 2017; Lucas et al. 2017). Thus, systems that orchestrate trade-offs can be mediated by a wide array of molecules. The trade-off might occur because of the pleiotropic effects of these chemical messengers and/or because these chemical messengers control behaviors that compete for the same pool of resources.

In addition to these neuroendocrine molecules, it is important to recognize a role for other molecules, such as sex determination factors, growth factors (e.g., transforming growth factor-β [TGF-β] and fibroblast growth factor-21 [FGF21]), prostaglandins, cytokines, (e.g., the proinflammatory cytokine, interleukin-6), and enzymes. Energy is the most important constraining factor that controls reproduction (Bronson 1989), and thus, it makes sense to include in this list the various inhibitors of the enzymes involved in intermediary metabolism, particularly glucose and fatty acid oxidation, which are central to energy allocation (Schneider et al.^1997^, ^1998^; Schneider and Zhou 1999). Energy for reproduction competes with energy for the immune responses, growth, and development, and chemical messengers such as FGF21 mediate the seasonal effects of day length on ingestive behaviors, energy storage, and energy expenditure (Samms et al. 2014, 2015), despite the fact that they do not technically qualify as hormones. Some enzymes and other chemical messengers not only partition energy toward ingestive behavior and away from reproduction, they literally decrease the oxidation of metabolic fuels and energy expenditure and increase the amount of energy that is shunted into storage in adipose tissue. When energy availability is limiting (low energy supply and high demand), these chemical messengers conserve energy that would be wasted on reproduction or wasted on mounting an immune response at a time when the energy must be expended on activities necessary for immediate survival (reviewed by Wade and Schneider 1992). In the first talk of our symposium, we learned that a small cluster of genes with pleiotropic effects on ingestive and reproductive behaviors has been discovered in *C. elegans.* At the time of this writing, *C. elegans* was the only species for which the entire connectome is known. The trade-off between uncertain reproductive success and assured survival in *C. elegans* is related to pleiotropic effects of the genes for some traditional endocrine receptors (e.g., insulin receptor, serotonin receptor, thyroid hormone receptor, and pigment dispersing factor) (Lipton et al. 2004; Barrios et al. 2012; Garrison et al. 2012; Emmons 2017)*.* In addition, this trade-off is influenced by sex determination factor, the vitamin D receptor, TGF-β, and the protein product of the *daf-12* gene. Some of these factors play a similar role in trading off sex for ingestive behavior in other model systems. (McCann and Hansel 1986; Wade et al. 1991; Heisler et al. 1994; Bruning et al. 2000; Sullivan et al. 2002; Benoit et al. 2004; Woodside et al. 2012; Zendehdel et al. 2012; Blevins and Ho 2013; Sabatier et al. 2013; French et al. 2014; Lopez-Esparza et al. 2015; Schellekens et al. 2015; Santoso et al. 2017; Woodside 2016). This initial 1-h kick-off to the symposium suggested many possibilities for linked genes with pleiotropic and/or antagonistic effects that would constrain phenotypic expression and lead to population divergence due to selection for different optima.

## The integration of physiology, brain, and behavior

Many effects of chemical messengers on behavior are assumed to occur in the brain; however, core elements of the trade-offs occur in peripheral tissues. The second talk scheduled in our symposium was to be given by Irene Miguel-Aliaga, but unfortunately, the talk was cancelled at the last minute due to unforeseen circumstances. It was to be a prime example of peripheral mechanisms involved in trade-offs. Miguel-Aliaga’s work is based on the fact that energetic investment in female reproduction includes hyperplasia of many tissues, including the midgut (intestines), which allows greater food ingestion and absorption of calories and nutrients. For example, in *Drosophila melanogaster*, females produce hundreds of eggs, and their fertility is enhanced by increased cell number and tissue size of the midgut. This remodeling of the digestive tissue is triggered by juvenile hormone, the secretion of which is stimulated by mating (Reiff et al. 2015). Thus, it appears that the nutrient allocation toward reproduction is not only dependent upon brain mechanisms that increase eating, but also peripheral mechanisms that increase the size of the gut in response to a reciprocal hormone-behavior relation. Specifically, the behavior of the mating pair elicits hormone secretion in the female, which enhances peripheral remodeling of the female digestive system, which in turn enables greater food ingestion and absorption (Reiff et al. 2015).

A broad, evolutionary overview of this topic in vertebrate and protovertebrate systems was provided in the presentation of George Bentley. He noted that the “neuroscience perspective” implicates the hypothalamus as the initiator of trade-offs between reproduction and survival when energy is constrained. Many of the so-called neuropeptides, however, are synthesized and released in the periphery, where they act on their cognate receptors to orchestrate trade-offs. For example, the gonadotropin-releasing hormone (GnRH) pulse generator has been assumed to be the locus of stress-induced, GnIH-mediated inhibition of reproduction, but in European starlings, *Sturnus vulgaris*, the gonads respond directly to fluctuations in corticosterone and metabolic fuels by increasing GnIH secretion and thereby modulating sex steroid secretion. Corticosterone upregulates GnIH expression in testes, whereas metabolic stress upregulates GnIH in ovaries, and modulation of gonadal secretion modulates reproductive and ingestive behavior (McGuire et al. 2013). In general, finding that an exogenous agent application to the brain elicits behavior does not exclude a role for this agent in peripheral control of cardiac, hepatic, enteric, digestive, excretory, and metabolic systems that feedback and control behavior. Similarly, it might be expected that interoreceptive and exteroreceptive signals that stimulate trade-offs in sex and ingestive behvior would be integrated within deep brain structures, but in *C. elegans*, the integration of sensory signals that determine the choice between food and sex is at least partially integrated at the premotor interneurons (Emmons 2017). This type of peripheral processing might be loosely analogous to the idea of “muscle memory.” Thus, an emerging theme of this symposium was the importance of the central and peripheral nervous system and its chemical and neural integration with peripheral tissues.

## Context is critical

Reproduction is more than just copulation and includes appetitive aspects of behavior, such as attractivity, mate searching, territoriality, courtship (including song), and maternal behaviors (Ferkin 2017). Ingestive behavior is more than just food intake per unit time, and includes the latency to eat, the rate of food consumption, food hoarding, and the preference for food versus opposite-sex conspecifics (Schneider et al. 2017). The symposium purposely included work on appetitive behaviors from many different species that represented most vertebrate taxa as well as some invertebrates. More than one group of researchers at this symposium emphasized the choice between food and sex as a phenotype in and of itself, and considered this approach more enlightening than studying daily food intake in the absence of mates or sex behavior in the absence of food (e.g., Emmons 2017; Lucas et al. 2017; Schneider et al. 2017). In addition, behavioral priorities are closely linked to prior energetic status, the presence of food, predators, and opposite-sex conspecifics. Genes, chemical messengers, and receptors were discovered by studying the option to engage in appetitive behaviors in their appropriate species-specific context. Appetitive behaviors are those that reflect motivation independent of performance (Craig 1917; Lorenz 1950). Attractivity is related to the ability to change the behavior of a potential mating partner (Beach 1976). Had some of these researchers restricted their attention to daily food intake in the absence of mating partners or copulation in the absence of food, the role of the various genes, chemical messengers, and receptors would not have been elucidated. Other talks illustrated that metabolic fuel availability and endocrine disrupting compounds have differential effects on sexual motivation, attractivity, and sexual performance (e.g., the contribution of Ferkin 2017). The mechanisms that govern the choice between food and sex are not just an interesting feature of one particular species. Rather, they may be a feature common to all living organisms, given that the choice between food and sex measured in different energetic conditions is a useful model system in mammals, reptiles, and nematode worms (Emmons 2017; Ferkin 2017; Lucas et al. 2017; Schneider et al. 2017). For example, Schneider et al. (2017) show that female hamsters are prodigious food hoarders in nature, but when females are fed unlimited amounts of food, they choose to spend time with males and ignore the opportunity to hoard food consistently on every day of the estrous cycle. Even ovariectomized females with little or no circulating ovarian steroids will visit males instead of hoarding food. By contrast, mildly food-restricted females become prodigious food hoarders on 3 out of the 4 days of the estrous cycle. Only on the day of estrous do they forgo food hoarding and visit males. Similarly, mildly food-restricted, ovariectomized females choose to hoard massive amounts of food rather than visit males, but treatment with estradiol and progesterone switch their priorities back to sex. The effects of ovarian steroids are masked in conditions of food abundance, and they are shown in sharp relief when energy availability is limited. Similarly, activation of RFRP-3 cells in the brain is increased by food restriction and this effect is modulated by ovarian steroids (Schneider et al. 2017). This theme was echoed in the contributions of Deviche in avian species and Bentley a wide array of chordates and protochordates (Bentley et al. 2017; Deviche et al. 2017). Context is important.

Demas emphasized the importance of context in species that forfeit reproductive opportunities in response to illness. Instead of searching and competing for mates, they often display sickness behaviors, such as fever, loss of appetite, and social isolation. Rather than simply indicating pathological side effects, sickness behaviors such as fever, loss of appetite, and social isolation can be viewed as coordinated adaptations that help fight infections and conserve energy (Hart 1988). Sickness behaviors and their effects on disease states vary with individuals and with environmental variables. Even the famous suppressive effects of gonadal steroids on immune function depend on reproductive context. Gonadal steroids and their receptors have a reputation for promoting reproductive success while simultaneously suppressing the immune system. In female rodents, however, testosterone lowers inflammation at ovulation, presumably preventing immune interference toward the sperm at conception. Thus, gonadal steroids can have positive influences on fertility while at the same time promoting an immune response (Lorenz et al. 2017).

The effects of environmental context on population divergence in heritable phenotypes are the essence of evolution by natural selection, and examination of hormone action in different environments can reveal either obligatory trade-offs or plasticity, which can avoid the need for trade-offs. For example, Crespi and Travis show that female killifish from populations at historically high densities are consistently smaller in size and give birth to small clutches of large offspring (Crespi and Travis 2017). Conversely, females from low-density populations with high predation risk are larger in size and give birth to large clutches of small offspring. These populations that developed in different environments also differ in their activity and mating behaviors exhibited in the presence of predators. For example, males from a high predation population exhibit high levels of female chase and lower levels of circulating cortisol after exposure to a predator than do males from a high density/low predation threat population. Conversely, reproductive output of females from the high density population is unaffected when housed in high density conditions, whereas those from the high predation/low density population dramatically reduce reproductive output when placed in high density conditions. In either case, prior exposure to stressors causes different reaction norms that favor higher reproductive output (e.g., adaptive plasticity). Thus, in this species and context, fitness trade-offs are not observed (Crespi and Travis 2017). Furthermore, levels of glucocorticoids and progestins vary between populations that were historically exposed to either high density or high predation risk, which might explain differences in the offspring size and number of trade-off exhibited by these populations (E. J. Crespi and J. A. Travis, unpublished data). The underlying mechanisms hypothesized by Crespi and Travis are new examples of the importance of context for hormonally-mediated trade-offs.

## Summary

One of the biggest draws of the annual meeting of the Society for Integrative and Comparative Biology is the agreement that an evolutionary and ecological context is critical for understanding underlying mechanisms. Conversely, molecular mechanisms inform our understanding of evolutionary processes. This symposium was a prime example of the excitement of integrating these two approaches.

Future challenges will include efforts to rigorously document the existence of trade-offs and flexible strategies. To discover the underlying molecular mechanisms, it will be important to move beyond the traditional endocrine mechanisms, now that we understand that growth factors, prostaglandins, and chemical messengers of the immune system can be involved in control of behavior and physiology. Furthermore, it will be important to consider the integration of central and peripheral systems, particularly metabolic systems. Both mainstream endocrinology and evolutionary endocrinology focus on hormones and their receptors. The question of how trade-offs arise from energetic constraints remains closely tied to unanswered questions about the effects of chemical messengers on metabolism, the nature and location of sensory detectors of metabolic fuel availability, and the effectors of fuel allocation and partitioning. In addition to application of the latest molecular techniques, it will continue to be important to understand the environmental/ecological contexts that lead to trade-offs and to use that understanding to design laboratory experiments that manipulate the relevant independent variables.

The research presented in this symposium is not only exciting, but also relevant to clinical/translational endocrinology. Most of the work presented at this symposium is funded by or potentially funded by the National Institutes of Health, the National Science Foundation, the United States Department of Agriculture, or other agencies that support research on climate change, conservation, and health and disease in people and domestic animals. For example, understanding the pleiotropic mechanisms that underlie the link between energy homeostasis, reproduction, aggression, stress, immunity, and longevity is relevant to understanding obesity, eating disorders, child abuse, infertility, inflammation, depression, and cancer in our own species (Korte et al. 2005). More specifically, Willis is making progress in understanding the role of energy constraints in trade-offs between immune function and homeothermy with regard to the epidemic of white nose syndrome in bats *M. lucifugus* (Willis 2017). Similarly, understanding the constraints and trade-offs associated with the evolution of sickness behaviors is relevant to drug development and therapeutic treatments for cancer, autoimmunue disease, and infections (Sylvia and Demas 2017). Clinical relevance and ecological/evolutionary importance are not mutually exclusive. Changes in climate and pollution directly impact health and disease as well as evolution and epigenetic change (Denver et al. 2009). Many of the contributors to this symposium are excellent examples of scientists who work without boundaries to link adaptive/maladaptive mechanisms to health and disease. They help us glimpse new ways to enrich and enliven our research programs by viewing the data within the context of integrative and comparative neuroendocrinology, ecology, and evolutionary biology.
